# Microwave-Assisted Synthesis of Iron-Based Aerogels with Tailored Textural and Morphological Properties

**DOI:** 10.1021/acsanm.3c04173

**Published:** 2023-09-27

**Authors:** Judith González-Lavín, Ana Arenillas, Natalia Rey-Raap

**Affiliations:** Instituto de Ciencia y Tecnología del Carbono, INCAR-CSIC, Francisco Pintado Fe 26, 33011 Oviedo, Spain

**Keywords:** iron oxide, sol–gel reaction, metallic nanostructured aerogels, microwave heating, designed properties

## Abstract

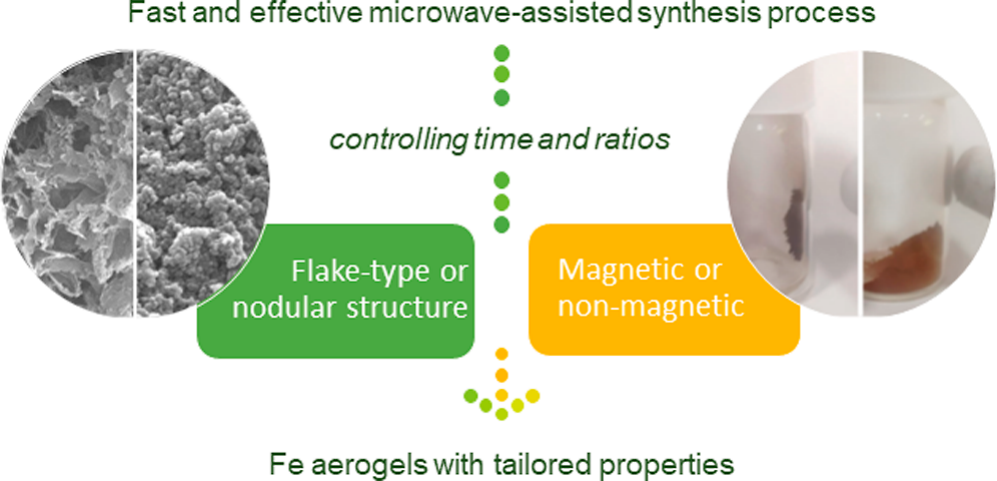

Iron aerogels have been synthesized by microwave heating for the first time. Therefore, it is essential to optimize this synthesis process to evaluate the possibility of obtaining nanometric materials with tailored properties and fitting them to the needs of different applications. Herein, the effect of the ratio between reagents and the time of synthesis on the final textural, morphological, and structural properties has been evaluated. The micro–meso–macroporosity of the samples can be tailored by modifying the ratio between reagents, whereas the time of synthesis has only a slight effect on the microporosity. Both the proportion between reagents and the time of synthesis are essential to controlling the nanometric morphology, making it possible to obtain either cluster- or flake-type structures. Regarding the chemical and structural composition, the samples are mainly composed of iron(II) and iron(III) oxides. However, the percentage of iron(II) can be modulated by changing the ratio between reagents, which implies that it is possible to obtain materials from highly magnetic materials to materials without magnetic properties. This control over the properties of iron aerogels opens a new line of opportunities for the use of this type of material in several fields of applications such as electrochemistry, electrocatalysis, and electrical and electronic engineering.

## 1 Introduction

Aerogels are nanomaterials obtained by the sol–gel method which form a continuous porous network with 90–99% of porosity.^1^ One of the great advantages of this synthesis process is that it allows tailoring the porosity and chemical properties of aerogels to fit the requirements of specific applications.^2^ This control can be performed by changing the synthesis conditions,^3,4^ specifically by modifying the nature and concentration of the precursors or the key steps of the process:^5,6^ (i) preparation of the sol, which involves the dispersion of the precursors; (ii) transition from sol to gel (known as gelation) in which cross-linking and branching between clusters take place, forming an interconnected chain structure; (iii) aging, which favors the increase of the backbone and mechanical strength of the structure; and (iv) drying, in which the solvent is removed from the pores of the aerogel. The conditions selected to perform these steps largely contribute to the final nanometric structure of the gel and hence to their physicochemical properties.^5,6^ Therefore, aerogels may exhibit, in addition to high porosity, different interesting properties such as high specific surface areas, low densities, high thermal insulation values, ultralow dielectric constant, and low refractive indexes.^1,6^

Regardless of the synthesis conditions, aerogels can be classified by the building blocks employed into three main groups: (i) molecular aerogels, which involve the use of alkoxides and hydrolysis and condensation reactions such as in the synthesis of SiO_2_, TiO_2_, Al_2_O_3_, or ZrO_2_ aerogels;^2,6,7^ (ii) polymer aerogels, which comprise the polymerization of monomers to obtain polymeric and carbon-derived aerogels like resorcinol-formaldehyde (RF), poly(vinyl alcohol), or melamine-formaldehyde aerogels;^2,6–9^ and (iii) colloidal aerogels, which involve the assembly of colloidal nanoparticles to obtain metallic aerogels.^10,11^ The most studied aerogel is based on silica, which was patented in 1918^12^ and reported by Kistler in 1932.^13^ Later on, in 1988, Pekala reported the first carbon aerogel based on RF.^14^ More recently, metallic aerogels based on noble metals have appeared and become popular due to their high oxidation resistance and catalytic activity.^10,15–19^ However, noble metals are scarce and costly, so in the last years, much effort has been made to replace them with transition metals (TMs).

Aerogels obtained from TMs (TMA) have been fruitful, although they were not straightforwardly synthesized until the 1990s. One of the TMAs that attracts the most interest is iron aerogels (FeAs) due to their magnetic properties, which have been promoted in recent decades due to their potential in fields such as medical diagnosis, catalysis, and sensors.^20^ The development of new synthesis methods to obtain FeAs was based on sol–gel methods previously studied in other systems, being necessary in many cases to use metal alkoxide compounds that are easily hydrolyzed.^21–24^ Unfortunately, the conventional synthesis of FeA via sol–gel processes often requires toxic chemicals at different stages of the reaction, such as epoxides or epoxide-based proton scavengers to induce gelation.^22,25–27^ In addition, long-lasting washing of the products, solvent-exchange processes, and post-treatments that can last for days are commonly required.^21,22,25–32^ On the other hand, another drawback of conventional FeA sol–gel synthesis routes is that the control of some properties such as crystallinity and magnetism is limited. Generally, amorphous structures are obtained that must be subsequently processed at high temperatures to achieve crystallinity.^24^ However, after these treatments, the porosity of the gel decreases considerably. Therefore, obtaining materials with high surface area and crystallinity as well as controlling their magnetic response is a major challenge. Therefore, new sol–gel strategies must be developed to shorten the process times via a simple and noncostly process, without resorting to toxic products, and able to control the porosity, crystallinity, and magnetism of the FeA.

Regarding the synthesis time, microwave (MW) technology has been proven to be sorely efficient in reducing the sol–gel reaction time in the synthesis of silica,^33–35^ carbon,^36–38^ and noble metal^39–41^ aerogels. Specifically, the use of MW in the synthesis of Pd aerogels allowed to reduce the reaction time from 24 to 7 h, maintaining the well-developed porous structure,^39^ demonstrating the potential of using MW heating for the synthesis of metallic aerogels. Nevertheless, the sol–gel synthesis of iron aerogels by MW heating has never been studied. In this context, it should be highlighted that in the sol–gel synthesis, the nature of the precursors is of paramount importance and makes it essential to optimize the synthesis process which involves parameters such as microwave conditions, volume of precursor solution, temperature, time, and reagents. The reaction mechanism of TMs, specifically iron, which could give rise to magnetic properties, is very different from that of materials such as silica gels, carbon gels, or noble metal aerogels; therefore, exhaustive studies are needed for each type of aerogel. Therefore, the novelty of this study lies in the development of the microwave-assisted synthesis of iron aerogels and the understanding of the synthesis route and the reaction mechanism that gives rise to different structures, which is key to being able to control the final properties. Therefore, the present work proposes the use of this heating technology to optimize the sol–gel process to obtain iron aerogels with controlled properties via a simple, quick, and nontoxic method.

## 2 Experimental Section

### 2.1 Precursor Solutions

Iron aerogels (FeAs) were synthesized by the sol–gel reaction using sodium carbonate (Indspec, 99%), glyoxylic acid (Sigma-Aldrich, 98%), and iron chloride (Sigma-Aldrich, 98%) as the main reagents and deionized water as a solvent. Initially, the reducing solution (solution R) was prepared by dissolving sodium carbonate (1.5 g Na_2_CO_3_) and glyoxylic acid (250 mg C_2_H_2_O_3_) in deionized water (250 mL). The metallic solution (solution M) was prepared by dissolving iron chloride (FeCl_2_) in deionized water with a concentration of 2 mg/mL. After dissolution, different precursor solutions (20 mL each) were prepared by mixing R and M with different ratios (R/M (v/v) = 4:1, 1:1, and 1:4), i.e., 4, 10, and 16 mL of R were mixed with 16, 10, and 4 mL of M, respectively. All preparations were carried out at room temperature.

### 2.2 Synthesis Conditions

**Scheme 1:**
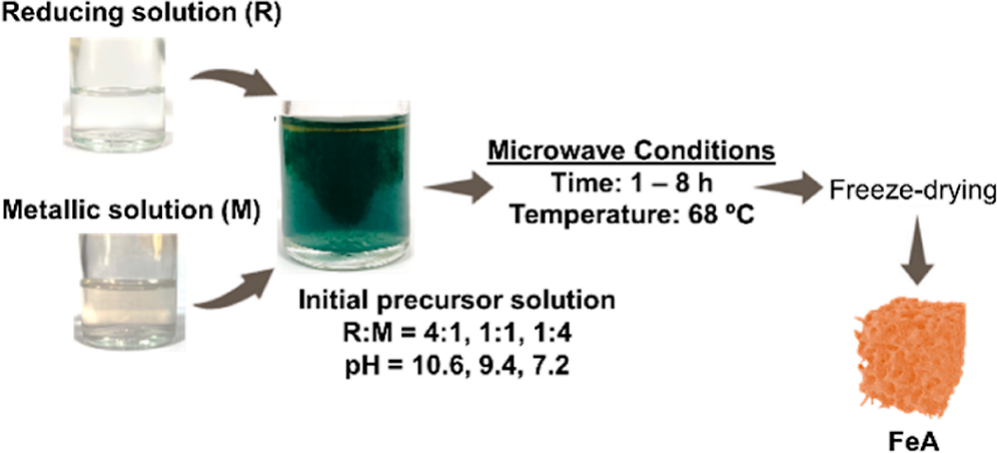
Illustration of the Microwave-Assisted Synthesis of Iron Aerogels (FeA)

Each precursor solution (20 mL) was introduced into a microwave system (Milestone ETHOS 1) to complete the reaction. The microwave employed was a multimode microwave reactor in which several Teflon vessels, which are hermetically sealed, can be irradiated at the same time. The device has a rotation system, which ensures homogeneous exposure of microwaves at all points and incorporates a controller to modulate the power of the magnetron. The temperature was kept constant at 68 °C, based on previous studies,^39^ and the reaction time was modified from 1 to 8 h. The temperature is monitored by a thermocouple, which is inserted directly into one of the vessels. After the reaction, the excess water was removed, and the precipitate was washed by centrifugation (3500 rpm, 5 min/cycle, and 5 cycles) to eliminate the unreacted products. Finally, the samples were frozen with liquid nitrogen and dried in a freeze dryer for 24 h to obtain the final FeA. The samples obtained were denoted as FeA-reaction time-R/M ratio. Thus, the sample FeA-1 h-4:1 corresponds to the ion aerogel obtained after 1 h of reaction with a reducing solution/metallic solution volume ratio of 4:1. Scheme 1 shows a brief description of the synthesis process.

### 2.3 Characterization Techniques

The porous properties of FeA were characterized by nitrogen adsorption/desorption isotherms on a Micromeritics Tristar II 3020 instrument. The samples were outgassed at 120 °C and 0.1 mbar using a Micromeritics VAcPrep 061 under vacuum for at least 12 h before performing the N_2_ isotherms. The specific surface area (*S*_BET_), the external surface area (*S*_ext_), and the micropore volume (*V*_DR_) were calculated by the Brunauer–Emmett–Teller (BET) equation, the *t*-plot method, and the Dubinin–Radushkevich method, respectively (more information regarding this technique is detailed in the Supporting Information). The DFT method applied to the adsorption branch, which takes into account the effects of surface roughness and heterogeneity, was used to obtain the pore size distributions (PSDs). The real density (ρ_He_) was measured in a helium pycnometry system (AccuPyc 1330 Micromeritics). The morphology was examined by scanning electron microscopy (SEM) using a Quanta FEG 650 microscope from FEI with an Everhart–Thornley detector (ETD). The samples were introduced into the microscope after being attached to an aluminum pin using conductive double-sided adhesive tape. An accelerating voltage of 20 kV and a spot size of 3 nm were used in all of the analyses. The SEM images were processed with the software ImageJ package to determine the size of the clusters that composed the aerogels. The microscope was coupled with an energy-dispersive X-ray analyzer Ametek-EDAX with an Apollo X detector to determine the chemical composition of the materials. The results represent the average values of each element detected at different points of the sample. The surface chemical composition was analyzed by X-ray photoelectron spectroscopy (XPS) using a Kratos AXIS Ultra HAS, with a monochromatic Al Kα X-ray source (1486.7 eV) with a pass energy of 30 eV for high-resolution regions of interest and 100 eV for the survey. The quantitative analysis was performed with the software CasaXPS applying the Shirley background. Raman spectra were recorded with a Jobin-Yvon LabRam HR UV 800 apparatus (Horiba Scientific) using a wavelength argon laser of 532 nm. Powder X-ray diffraction (XRD) was performed using Cu Kα radiation on a D8 ADVANCED diffractometer to study the phase structure and crystallinity of the compounds. The data was collected over a 2θ range from 30.00 to 65.00° with a step size of 0.05°. The identification of the crystalline phases was carried out using Diffrac EVA software.

## 3 Results and Discussion

**Figure 1:**
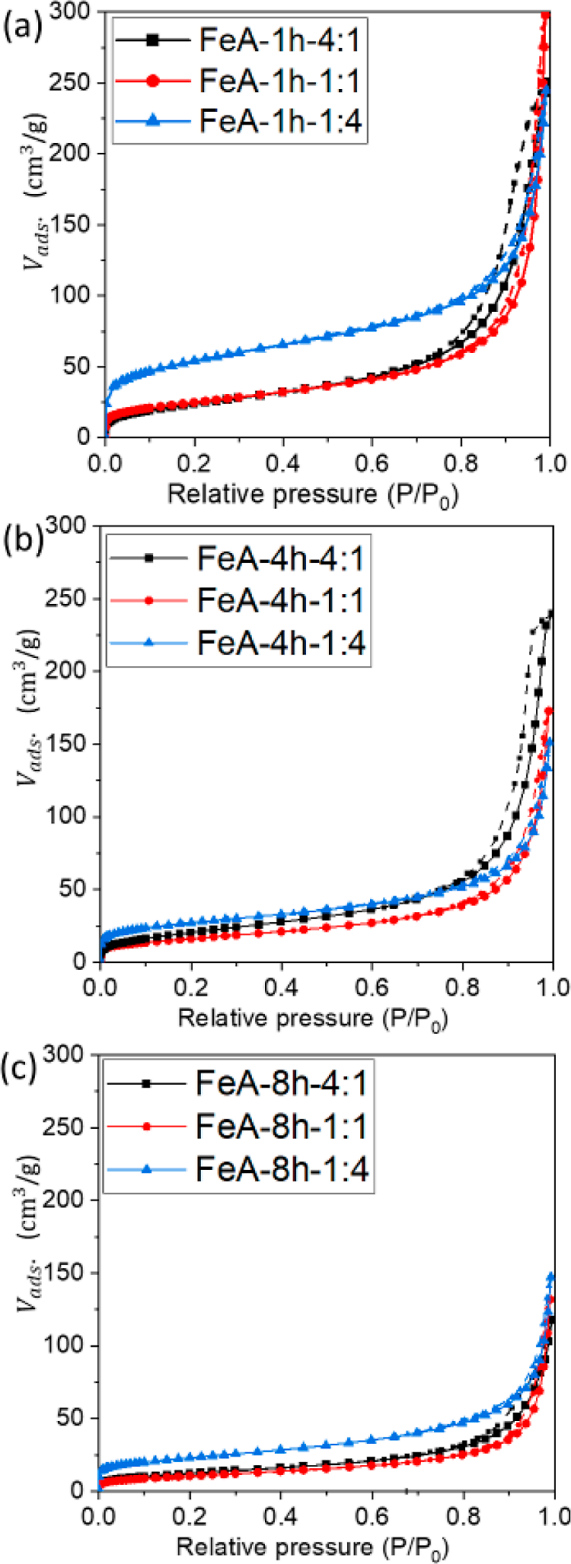
Nitrogen adsorption–desorption isotherms of iron aerogels synthesized at (a) 1, (b) 4, and (c) 8 h with different R/M ratios 4:1 (black), 1:1 (red), and 1:4 (blue).

Nitrogen adsorption–desorption isotherms were performed to evaluate any possible modification of the textural properties of FeA due to variations in the synthesis conditions. The isotherms obtained and the most important textural parameters are shown in Figure 1 and Table 1, respectively. The shape of the isotherms varies according to the R/M ratio and the synthesis time, which suggests that different porous structures can be obtained by modulating the synthesis conditions. Besides, some of the isotherms present a hysteresis loop, whose formation is associated with capillary condensation processes that take place due to the filling of the mesopores and the subsequent evaporation processes. Generally, the latter takes place at a pressure lower than that of capillary condensation, giving rise to the formation of the hysteresis loop. These processes depend on the shape of the mesopores, so the shape of the hysteresis loop can give valuable information regarding mesoporosity. In this context, the isotherm of sample FeA-1 h-4:1 can be classified as Type IV according to the IUPAC classification, exhibiting an H3 hysteresis loop. This type of isotherm can be attributed to mesoporous materials with wedge-shaped pores,^42^ which is confirmed by the PSD shown in Figure S1. Comparing the series of samples FeA-1 h-R/M (Figure 1a), it can be observed that the hysteresis loop decreases as the proportion of metal precursor increases, resulting in Type II isotherms related to macroporous materials.^42,43^ This phenomenon can be due to the distribution of clusters that form the aerogel. The higher the concentration of metal precursor, the larger the number of nucleation points, and hence, a larger number of clusters are formed. These clusters branch together forming a longer chain, leaving a broader space between them, i.e., macropores. The shape of the hysteresis loop of the samples prepared for 4 h (Figure 1b) and 8 h (Figure 1c) follows the same trend as those synthesized for 1 h (Figure 1a), suggesting, once again, that increasing the proportion of the metal precursor results in porous structures that evolve from mesoporous to macroporous materials. This evolution is confirmed by the PSD shown in Figure S1. These results are in agreement with the density values, which decrease with an increase in the proportion of the metal precursor (5.5, 5.0, and 3.6 g/cm^3^ for the series of samples FeA-*t*-4:1, FeA-*t*-1:1, and FeA-*t*-1:4, respectively).

**Table 1:** Porous Properties Obtained from the N_2_ Adsorption–Desorption Isotherms

sample	BET (m^2^/g) ± 2	*S*_ext_ (m^2^/g) ± 1	*V*_DR_ (cm^3^/g) ± 0.01
Fe-1 h-4:1	74	74	0.03
Fe-1 h-1:1	76	73	0.03
Fe-1 h-1:4	187	193	0.08
Fe-4 h-4:1	72	71	0.03
Fe-4 h-1:1	57	47	0.02
Fe-4 h-1:4	80	78	0.04
Fe-8 h-4:1	45	41	0.02
Fe-8 h-1:1	37	33	0.01
Fe-8 h-1:4	85	83	0.04

Regarding the microporosity, the volume adsorbed at low relative pressure increases with the proportion of the metal precursor for those samples synthesized for 1 h, indicating an increase in the volume of micropores. Once again, this effect can be due to the formation of a larger number of clusters as microporosity is generated inside the clusters. The microporosity of sample FeA-1 h-4:1 is similar to that of FeA-4 h-4:1, suggesting that the synthesis time does not affect its microporous structure. Contrarily, differences in the BET surface area are observed for those samples synthesized with 1:1 and 1:4 ratios, whose values decrease by increasing the synthesis time. This effect is diminished as time increases from 4 to 8 h. Regarding the external surface area (*S*_ext_), this parameter follows the same trend as the BET surface area. From these results, it can be inferred that there is a notable modification in the porous structure as a function of the R/M ratio.

**Figure 2:**
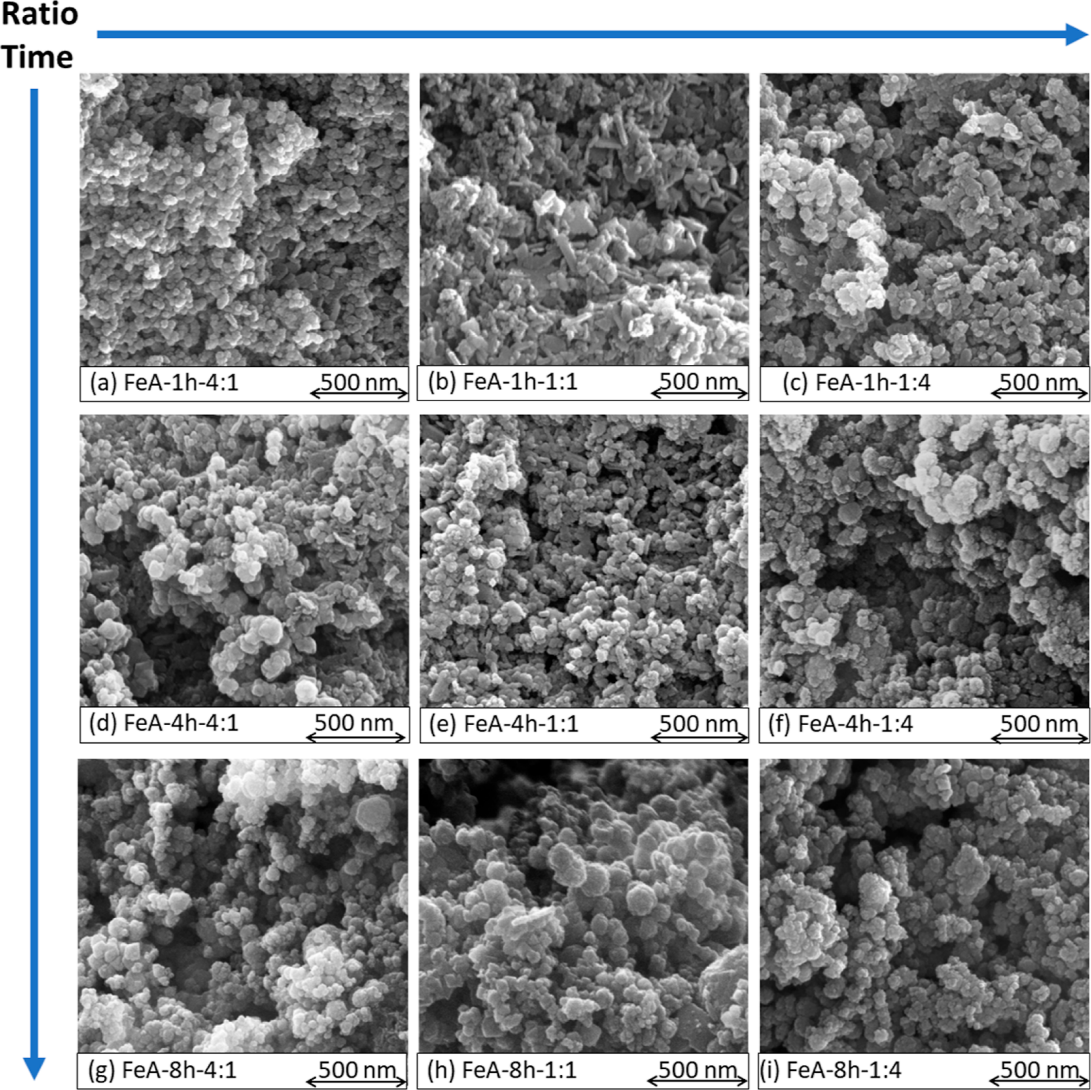
SEM images of the FeA synthesized for 1 h with ratios R/M (a) 4:1, (b) 1:1, and (c) 1:4; 4 h with ratios R/M (d) 4:1 and (e) 1:1; and (f) 1:4 and 8 h with ratios R/M (g) 4:1, (h) 1:1, and (i) 1:4.

To further understand the effect of the synthesis process, we evaluated the morphology of the samples by SEM. The nanometric morphology of the aerogels synthesized from precursor solutions with different ratios between regents (R/M) and different synthesis times is shown in Figure 2. Regardless of the time of synthesis, a cluster-type structure is formed by using the R/M ratios 4:1 (Figure 2a,d,g) and 1:4 (Figure 2c,f,i), while with a ratio of 1:1, a flake-type structure appears (Figure 2b,e,h), especially after only 1 h of reaction. The differences between these two types of structures are shown in more detail in Figure S2, in which images taken at higher magnitudes are shown.

**Figure 3:**
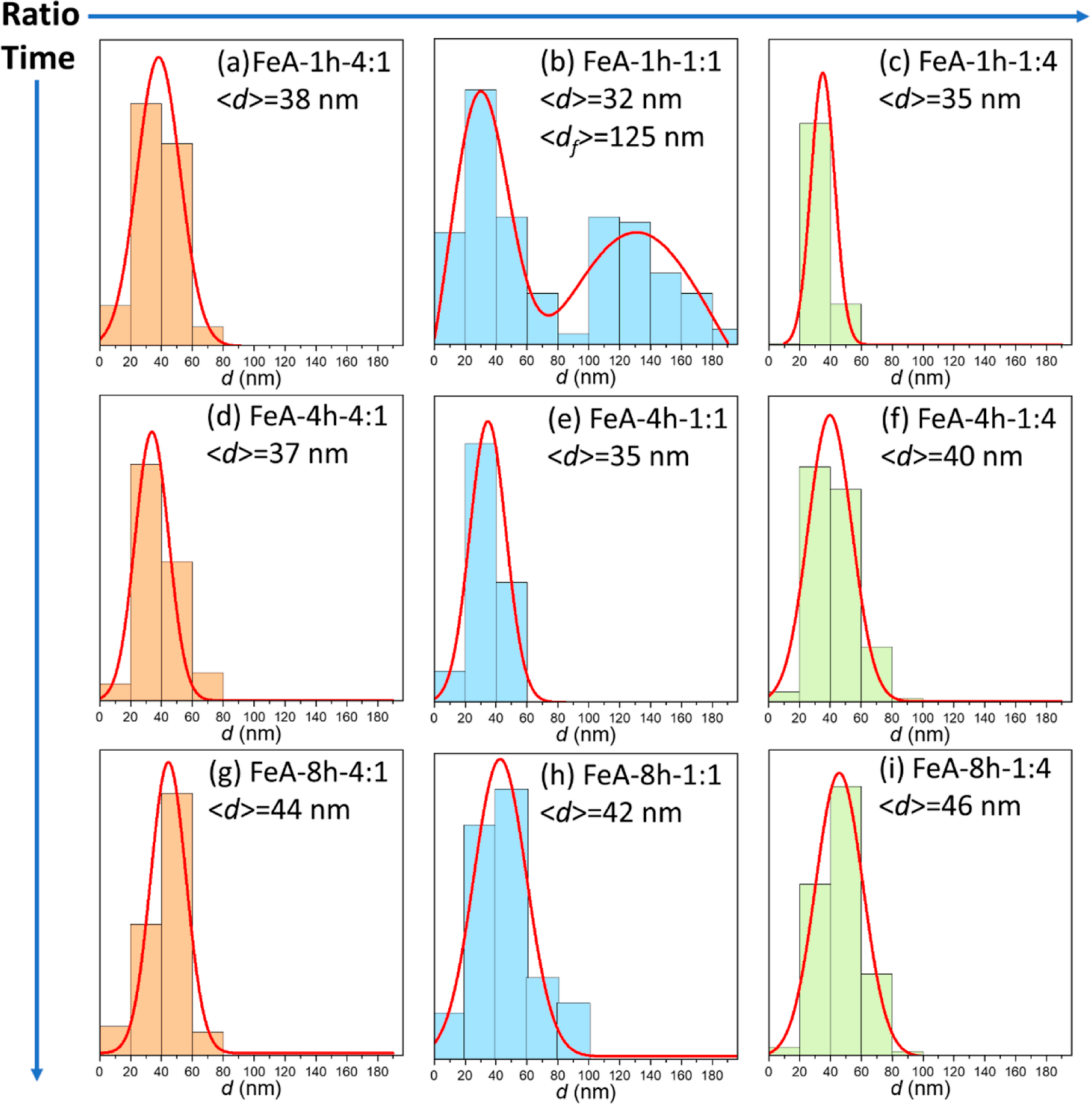
Histograms of the aerogels synthesized for 1 h with ratios R/M (a) 4:1, (b) 1:1, and (c) 1:4; 4 h with ratios R/M (d) 4:1, (e) 1:1, and (f) 1:4; and 8 h with ratios R/M (g) 4:1, (h) 1:1, and (i) 1:4.

SEM images showed the differences in the size of the nanoclusters due to the modification of the ratio R/M and time of synthesis. The size distribution was determined by evaluating at least 6 different SEM images of each sample, and the collected data were fitted using Gaussian functions (Figure 3).

The results show that the average cluster sizes of the samples prepared with ratios 4:1 and 1:4 are very similar. However, the cluster size increases with time, demonstrating that there is a direct relationship between them. The behavior of the samples synthesized with an R/M ratio of 1:1 is completely different. In this case, a nanometric flake-like structure predominates when the reaction time is short (1 h), with few clusters scattered in the network forming the flakes (Figures 2b and S2). The appearance of these two different structures is also evidenced in the cluster size distribution shown in Figure 3b, as two Gaussian functions are needed to fit it: one attributed to the clusters (*d*) and the other to the flakes (df, measured longitudinally). However, the number of clusters increases with the time of synthesis, until achieving a structure mainly composed of clusters (Figure 2h) with an average size close to 42 nm (Figure 3h), which is similar to that observed for the other two samples prepared for 8 h (Figure 3g,i).

**Figure 4:**
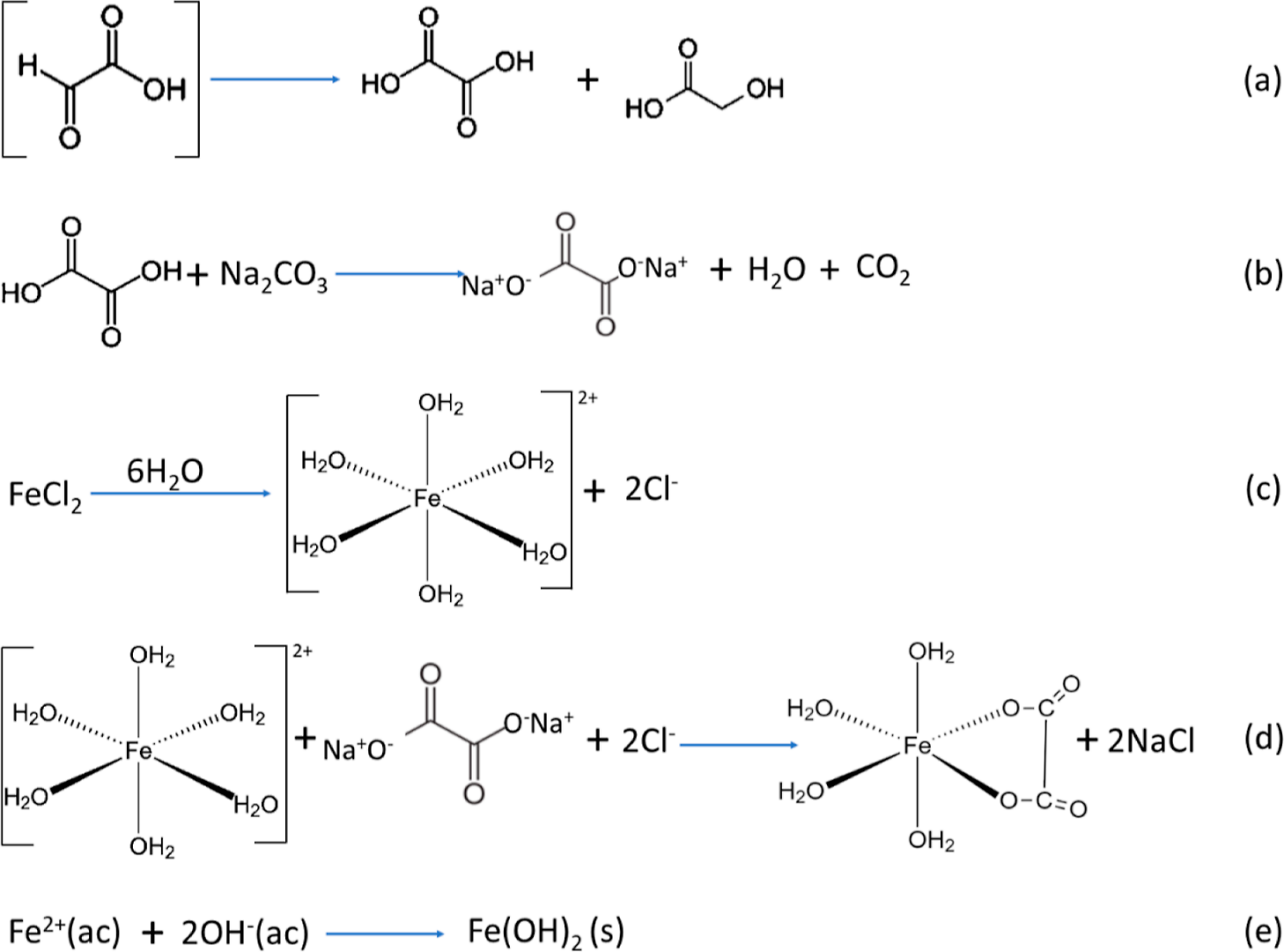
Step-by-step process of the reaction mechanism of (a) glyoxylic acid to oxalic acid; (b) reaction between oxalic acid and sodium carbonate; (c) solvation of iron ions in aqueous media; (d) reaction between 6-hydroxoferrate(II) and oxalate ions; and (e) precipitation of iron ions in the form of Fe(II) hydroxide.

To understand the morphological differences of iron aerogels depending on the synthesis conditions, it is essential to take a deep view of the chemical reactions that take place during the sol–gel process. As explained in the Experimental Section, the aerogels were obtained by mixing a reducing solution (R) with the metallic precursor (M). The preparation of the reducing solution (composed of sodium carbonate and glyoxylic acid) in basic media gives rise to the deprotonation of the glyoxylic acid, resulting in a transparent solution (Figure S3a) with a pH of 10.4 composed of oxalic and glycolic acid, as shown in Figure 4a. Then, an acid–base reaction occurs between the oxalic acid and the sodium carbonate, giving rise to an oxalate that conjugates with the sodium cation, since carbonate is unstable to proton charge and forms a dicarboxylate, as shown in Figure 4b. On the other hand, the metallic precursor was prepared by dissolving iron chloride in water. In aqueous media, the iron ions are solvated following the reaction shown in Figure 4c. The color of this solution is slightly orange (Figure S3b) and has a pH value of 3.8. Fe^2+^ is a Lewis acid and has a low electron density; therefore, the surrounding water molecules are able to share some of their charge density, releasing relatively acidic protons into the medium. This transfer of protons produces the hydroxide ligands (6-hydroxoferrate(II)), which are placed in the form of an octahedral complex.^44^

Once the reducing solution and the metallic precursor are mixed, the oxalate ions displaced the water molecules coordinated with the iron, resulting in coordination between the oxalate and the iron (Figure 4d). Simultaneously, precipitation of part of the iron ions as Fe(OH)_2_ occurs due to the basic pH of the medium (pH = 10.6, 9.4, and 7.2 for ratios 4:1, 1:1, and 1:4, respectively), Figure 4e. The electronic transitions between the iron and the ligand, together with the precipitation of Fe(OH)_2_, contribute to the production of insoluble green complexes, referred to in the literature as green rusts I (GRI)^44–46^ (Figure S3c). The appearance of GRI is immediate, as all the reactions detailed in Figure 4 are very fast.

**Figure 5:**
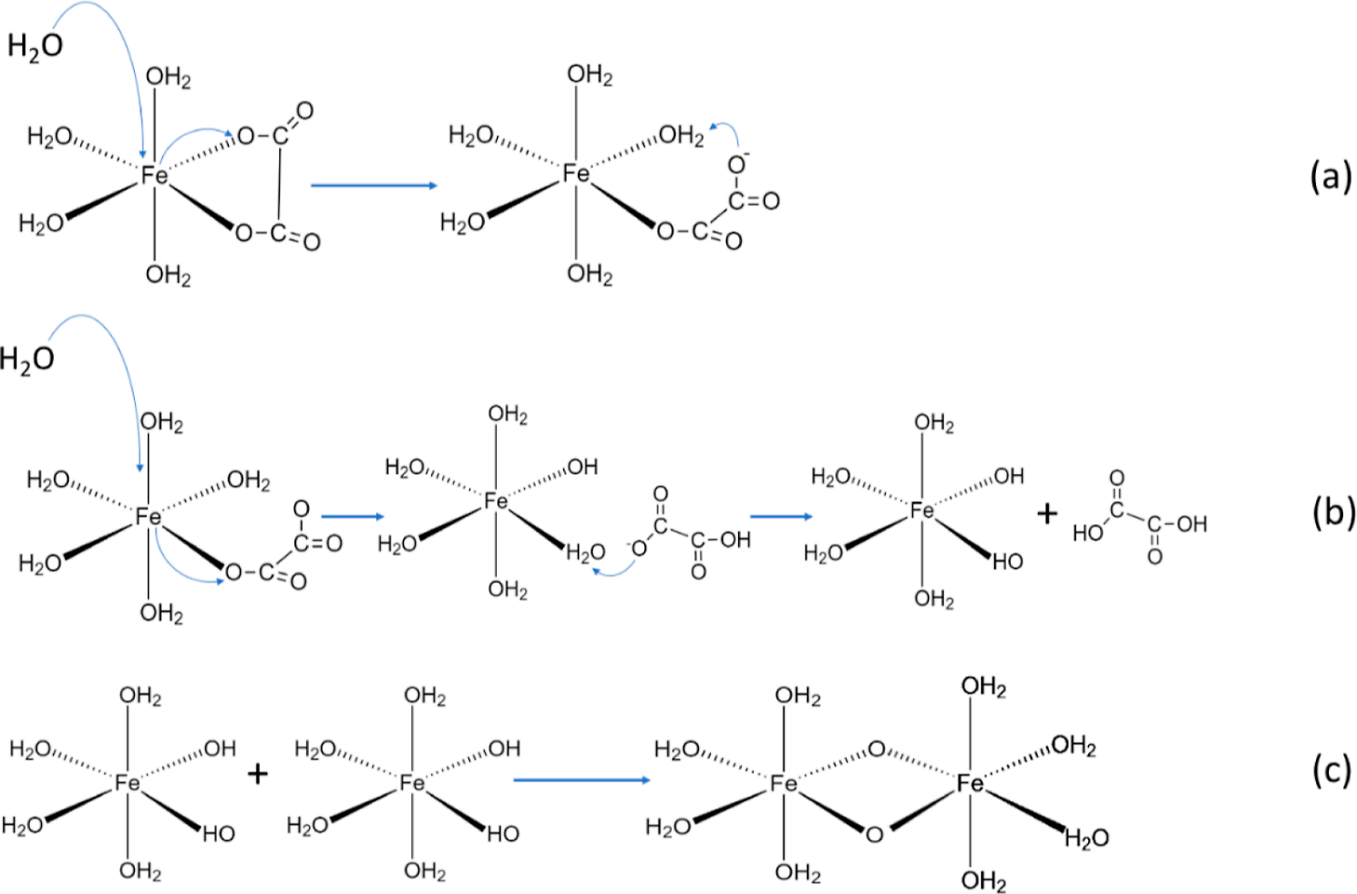
Steps of the sol–gel process that take place in the synthesis of iron aerogels: nucleophilic reaction (a), olation/oxylation reactions (b), and particle aggregation (c).

After the formation of the GRI, this solution is introduced in the microwave oven to promote the reaction, which starts at the nucleation points, i.e., the GRI. Three stages can be defined in a regular sol–gel process under basic conditions to obtain metallic aerogels: (i) nucleophilic attack to the metallic atom through the oxygen present in a water molecule, (ii) the proton transfer from a water molecule to a –OR group from the metal (in the case of the synthesized FeA, R is the oxalate), and (iii) the liberation of ROH molecules^47–49^ (Figure 5a). Once the hydroxyl groups coordinated with the cation start to condense with another group, two mechanisms are concatenated: olation and oxylation. The olation takes place when the metallic atom coordination number is not full, and hydroxyl bonds appear from the initial hydroxyl groups. This is a nucleophilic addition reaction with fast kinetics, which does not carry any other change in the coordination sphere. As the reaction progresses and the structure grows, oxylation starts, forming oxygen links between metallic atoms and giving rise to oxolate species, which are the crystallization points (Figure 5b). The kinetics of the oxylation is slower than the kinetics of the olation because a nucleophilic substitution is required and implies the elimination of a water molecule. After successive stages of olation/oxylation, the crystallization points grow into primary particles that aggregate (Figure 5c),^46,50–54^ resulting in an inorganic network. Due to the high molecular weight of iron, the precipitate falls as a dark solid.

The kinetics of the olation and oxylation reactions depends on the proportion of the reagents, i.e., the R/M ratio employed. In the case of R/M ratios of 4:1 and 1:4, in which there is always an excess reagent, the olation is fast enough to reach the nucleophilic substitution during the synthesis, resulting directly in the formation of clusters. However, the olation reaction becomes the limiting step with an R/M ratio fixed at 1:1. Therefore, the spatial disposition of the links gives rise to the formation of layers, reaching the flake-type structure (Figure S2b,e). By increasing the reaction time, olation is accomplished and oxylation takes place, resulting in the nucleophilic substitution and formation of oxygen links between metallic atoms and, hence, in the formation of clusters (Figure S2). The probability that the substituted bonds in the iron complex will not be equatorial may increase with time, thus forming three-dimensional structures.

The chemical composition of those samples synthesized for 4 h was analyzed by using EDX. Iron, oxygen, and carbon were detected in all of the samples at different points, while no traces of impurities were detected. The percentage of carbon was close to 2 wt % for all the samples, while the percentage of oxygen increased with the percentage of metal precursor (3, 8, and 15 wt % for samples Fe-4 h-4:1, Fe-4 h-1:1, and Fe-4 h-1:4, respectively). This increase is accompanied by a decrease in the percentage of iron from 96 to 83 wt %, suggesting that the percentage of oxidized iron species increases with the percentage of metallic solution in the precursor mixture.

**Figure 6:**
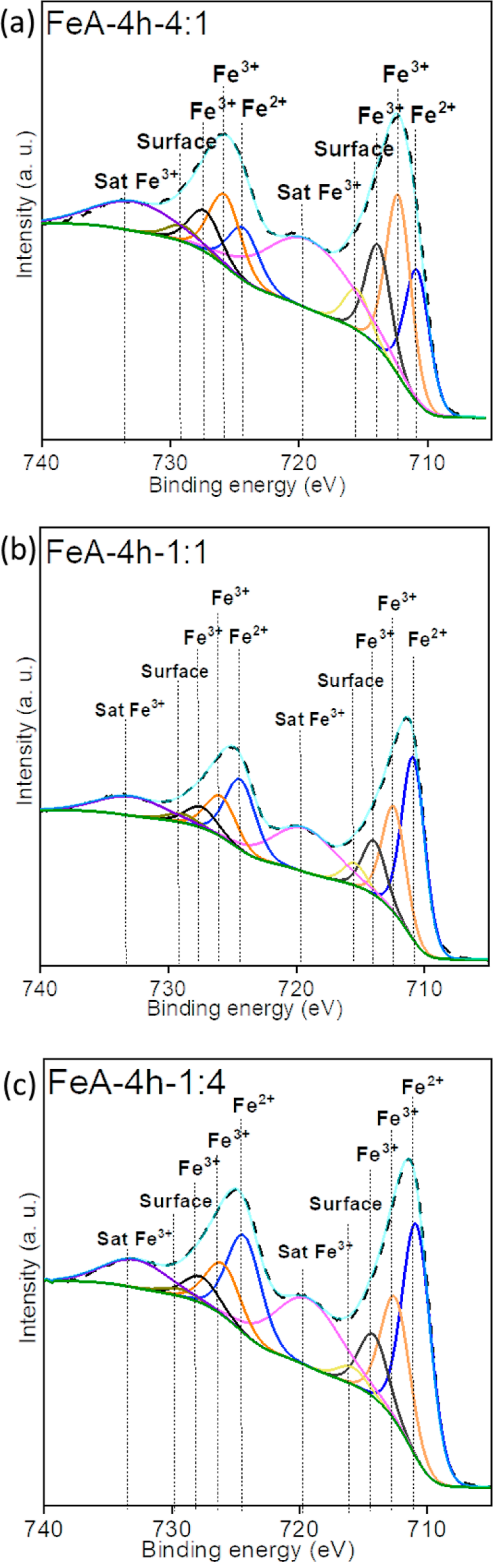
XPS spectra of the iron Fe 2p region for the samples (a) FeA-4 h-4:1, (b) FeA-4 h-1:1, and (c) FeA-4 h-1:4.

The chemical surface composition of the iron aerogels was also analyzed. The presence of iron, oxygen, and carbon was verified by XPS analysis (Figure S4), which agrees with the results obtained by EDX. The chemical state of iron over the surface of the aerogels was evaluated by analyzing the high-resolution region of Fe 2p. The high-resolution XPS spectra in the Fe 2p region were deconvoluted into 10 peaks,^55^ as shown in Figure 6; 5 of them within the region centered at 711 eV and the other 4 within the region centered at 724.5 eV, each region attributed to Fe 2p_3/2_ and Fe 2p_1/2_, respectively. The five peaks in Fe 2p_3/2_ are shown at 710.8 ± 0.1 eV (Fe^2+^), 712.4 ± 0.1 eV (Fe^3+^), 714.0 ± 0.2 eV (Fe^3+^), 715.6 ± 0.2 eV (surface peak), and 719.3 ± 0.1 eV (Fe^3+^ satellite peak, Fe_2_O_3_).^56^ Each of these peaks presents the corresponding spin–orbit coupling with a shift in the binding energy of 13.5 eV (Fe 2p_1/2_ in the 724.5 eV region). These results suggest that all samples are composed of a mixture of iron oxides [Fe(II) and Fe(III)]. The position, fwhm, and percentage of each iron species in Fe 2p_3/2_ can be found in Table S1. These data suggest that the percentage of Fe(II) increases with the amount of metallic precursor (19, 38, and 40% for samples FeA-4 h-4:1, FeA-4 h-1:1, and FeA-4 h-1:4, respectively). Besides, a slight decrease is observed by increasing the time of synthesis (42 and 40% for samples FeA-1 h-1:4 and FeA-4 h-1:4, respectively), indicating that the chemical structure of the FeA can be tailored by controlling the synthesis conditions.

**Figure 7:**
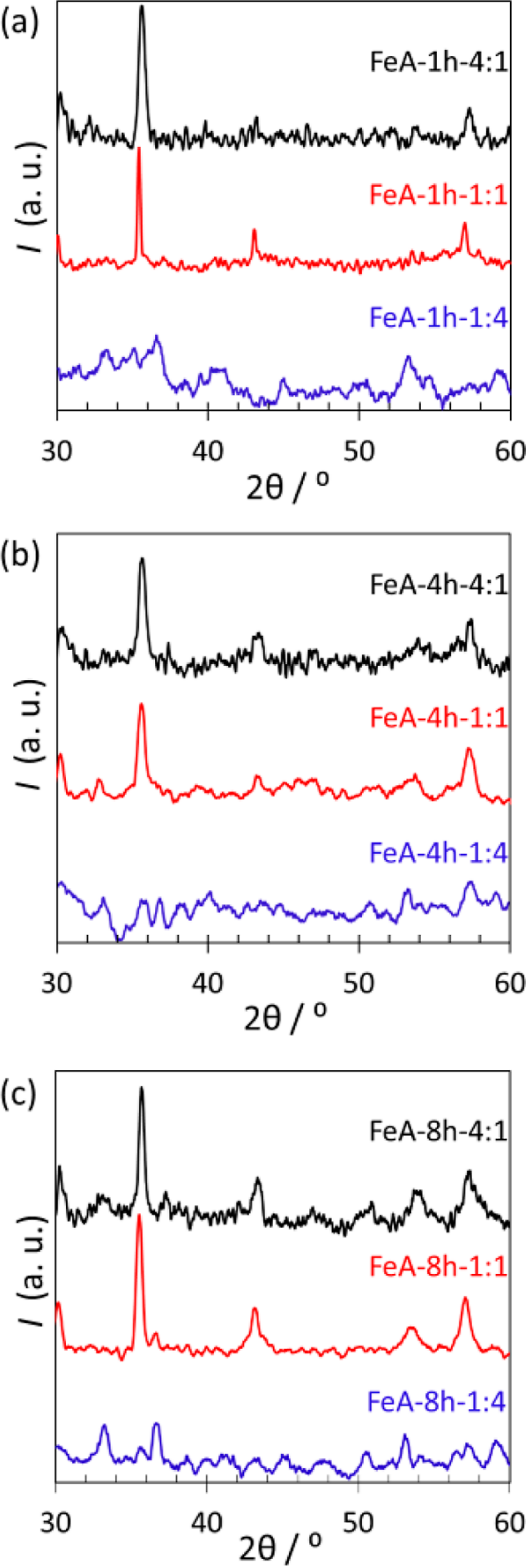
XRD pattern for the samples synthesized for (a) 1, (b) 4, and (c) 8 h for the three studied R/M ratios: 4:1 (black), 1:1 (red), and 1:4 (blue).

Compositional results and the crystalline phase of all of the samples were evaluated by XRD to obtain more information about the iron oxides formed by modifying the R/M ratio and synthesis time. The spectra obtained are shown in Figure 7. The XRD profile of the FeA powder was composed of broad lines related to an amorphous structure. However, there are crystalline structures within the clusters of some materials, whose signals were high enough to identify the iron oxide phases. The presence of carbon-containing phases, such as carbonates, was not observed. This element may be part of the amorphous structure responsible for the noise in the signal.

Comparing the series of samples FeA-1 h-R/M (Figure 7a), FeA-1 h-4:1, and FeA-1 h-1:1 show the strongest and clearest peaks. These peaks produced by constructive interference with the crystalline planes appear at 35.4, 43.0, 53.4, and 56.9° attributed to the (311), (400), (422), and (511) diffraction planes (*hkl*), respectively. These planes can be related to the reference standards of maghemite and magnetite, whose patterns (Figure S5) are equal and, hence, make it difficult to distinguish between both structures. The XRD patterns of the series of samples FeA-*t*-1:4 do not present any relevant crystalline peak, suggesting that the samples are mostly composed of amorphous iron oxide structures, involving Fe(II) and Fe (III) as analyzed in XPS. The diffractogram patterns of the samples prepared for 4 h (Figure 7b) and 8 h (Figure 7c) follow the same trend as those synthesized for 1 h (Figure 7a), suggesting that, regardless of the synthesis time, increasing the proportion of the metal precursor results in more amorphous structures. This effect suggests an evolution of the synthesis route, probably related to the pH of the initial precursor solution. For ratios 4:1 and 1:1, the basic nature of the precursor solution (pH values of 10.4 and 9.4) may result in a coprecipitation process, followed by a later aggregation of nanocrystals leading to the crystalline phase detected by XRD. On the other hand, the initial precursor solution of sample FeA-*t*-1:4 was ca. 7.2, which favored the formation of amorphous sol–gel structures. The crystallite size of the series of samples FeA-*t*-4:1 and FeA-*t*-1:1 was estimated from the XRD peak at 2θ of 36° using the Scherrer formula represented by eq S1.^57^ The estimated crystallite sizes are detailed in Table S2. All the analyzed samples show crystallites within the nanoscale, but interesting differences can be observed. Regardless of the time of synthesis, the crystallite size of sample FeA-*t*-4:1 is smaller than that of sample FeA-*t*-1:1 (Table S2), which is in agreement with the above-mentioned hypothesis about the influence of the pH, i.e., the more basic the precursor solution, the higher the degree of crystallinity in the structure. Besides, the crystallite size decreases by increasing the synthesis time, probably due to aggregation effects, which are favored over time. These results indicate that the crystallinity of these compounds highly depends on the synthesis conditions.

Aside from crystallinity, the series of samples FeA-*t*-4:1 and FeA-*t*-1:1 exhibit a magnetic response (Figure S4a,b,d,e,g,h) and a brownish color, which suggests that these aerogels may be composed of magnetite and magnetite, which commonly presents ferromagnetic properties and a dark color. Contrary, the series of samples FeA-*t*-1:4 have a small or even no magnetic response (Figure S4c,f,i) and a more reddish color. This is in agreement with those results obtained by XRD, in which it was observed that this series was composed of amorphous iron oxide structures different from maghemite/magnetite.

## 4 Conclusions

Iron aerogels were synthesized by a sol–gel reaction assisted by microwave heating. Different samples were prepared by changing the proportion between reagents (reduction and metallic solution) and the synthesis time. It was found that the ratio between reagents modifies the textural properties, as the materials evolve from mesopore to macropore structures, and the microporosity increases by increasing the proportion of the metallic solution. This chemical variable also modifies the nanometric morphology of the samples. The SEM images suggest that cluster-type structures are favored by the increase in the proportion of the reduction solution or metallic solution, whereas a flake-type structure appears for aerogels prepared with equal volumes of reagents. These differences in morphology are related to the chemical reactions that take place during the synthesis process: olation and oxylation. An excess of reagents favors the kinetics of the olation, so the reaction is limited by the oxylation, which gives rise to cluster-type structures. Contrarily, the kinetics of the olation is decreased by using equal volumes of reagents, resulting in the formation of flake-type structures. However, an increase in the synthesis time results in the formation of clusters as sufficient time is given for oxylation to take place. EDX shows that there are more oxidized species as the amount of initial metal precursor increases, while the analysis of the surface chemical composition shows that FeA are mainly composed of different iron oxides, increasing the amount of Fe(II) by increasing the proportion of the metallic precursor. On the other hand, the XRD patterns show that the degree of crystallinity increases by decreasing the proportion of the metallic precursor, probably due to the basic nature of the initial precursor solution that results in crystalline structures. Besides, the higher the crystallinity of the structure, the higher the magnetic response of the final samples. All of these results indicate that the microwave-assisted synthesis of FeA allows the control of the final textural, morphological, and structural properties by modifying the synthesis conditions. This is presented as an interesting advantage in the field of TM aerogels as they can be optimized for a large number of applications such as electrocatalysts in electrolyzers and fuel cells or reduction of CO_2_ and electrode materials in batteries and sensors or electromagnetic absorption materials.

## Data Availability

All data needed to evaluate the conclusions in the paper are present in the paper and/or the Supporting Information document. Additional raw/processed data related to this paper may be requested from the authors.

## Supporting Information Available

PSDs obtained from the DFT method; SEM images; photographs of the reducing, metallic, and precursor solutions; XPS survey; deconvolution of the high-resolution XPS spectra in the Fe 2p_3/2_ region; PDF standard XRD patterns; characteristic parameters obtained from XRD using the Scherrer equation; and photographs of the samples’ magnetic response (PDF)
