# Supplementary material for: Microwave-Assisted Synthesis of Iron-Based Aerogels with Tailored Textural and Morphological Properties
Source: ACS Appl Nano Mater. 2023 Sep 27;6(19):18582–91. doi: 10.1021/acsanm.3c04173 (PMC10580704; doi:10.1021/acsanm.3c04173)
Supplement: Supplementary file 1 — an3c04173_si_001.pdf [file an3c04173_si_001.pdf]

SUPPORTING INFORMATION

Microwave-Assisted Synthesis of Iron-Based Aerogels with Tailored Textural and Morphological Properties

Judith González-Lavín, Ana Arenillas, Natalia Rey-Raap*

Instituto de Ciencia y Tecnología del Carbono*, INCAR-CSIC, Francisco Pintado Fe 26, 33011 Oviedo, Spain*

*[*natalia.rey@incar.csic.es](mailto:natalia.rey@incar.csic.es)*

Abstract

Iron aerogels have been synthesized by microwave heating for the first time. Therefore, it is essential to optimize this synthesis process to evaluate the possibility of obtaining nanometric materials with tailored properties and fit them to the needs of different applications. Herein, the effect of the ratio between reagents and the time of synthesis on the final textural, morphological, and structural properties has been evaluated. The micro-meso-macroporosity of the samples can be tailored by modifying the ratio between reagents, whereas the time of synthesis has only a slight effect on the microporosity. Both the proportion between reagents and the time of synthesis are essential to control the nanometric morphology, making it possible to obtain either cluster or flake-type structures. Regarding the chemical and structural composition, the samples are mainly composed of iron(II) and iron(III) oxides. However, the percentage of iron (II) can be modulated by changing the ratio between reagents, which implies that it is possible to obtain from highly magnetic materials to materials without magnetic properties. This control over the properties of iron aerogels opens a new line of opportunities for the use of this type of materials in several fields of applications such as electrochemistry, electrocatalysis, and electrical and electronic engineering.

Textural Properties

The textural properties of the FeA were characterized by nitrogen adsorption/desorption isotherms on a Micromeritics Tristar II 3020 instrument. The samples were previously outgassed at 120 °C and 0.1 mbar using a Micromeritics VAcPrep 061 under vacuum for at least 12h. The specific surface areas (S_{BET}) were calculated by the Brunauer–Emmett–Teller (BET) equation applied to the nitrogen adsorption branch in a linear region within the relative pressure range of 0.03−0.132 following Rouquerol method. The micropore volumes (V_{DR}) were determined using the Dubinin–Raduskevich method, while the external surface area (S_{ext}) was obtained by applying the t-plot method. The pore size distributions obtained from the DFT method applied to the adsorption branch of the nitrogen isotherms are shown in Figure S1. Regardless of the synthesis time, the FeA samples evolve from mesoporous to macroporous materials by increasing the proportion of metal precursor (from 4:1 to 1:4).

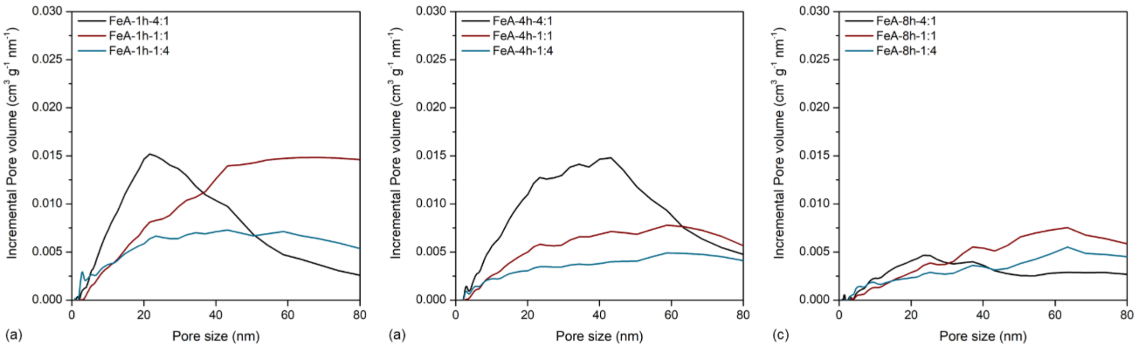

Figure S1. Pore size distributions obtained from the adsorption branches of the isotherms by the DFT method.

Morphological Properties

The morphology of the samples FeA-1h-R:M evaluated by scanning electron microscopy (SEM) are shown in Figure S2. A cluster-type structure is formed by using the R:M ratios 4:1 and 1:4, while with a ratio of 1:1, a flake-type structure appears.

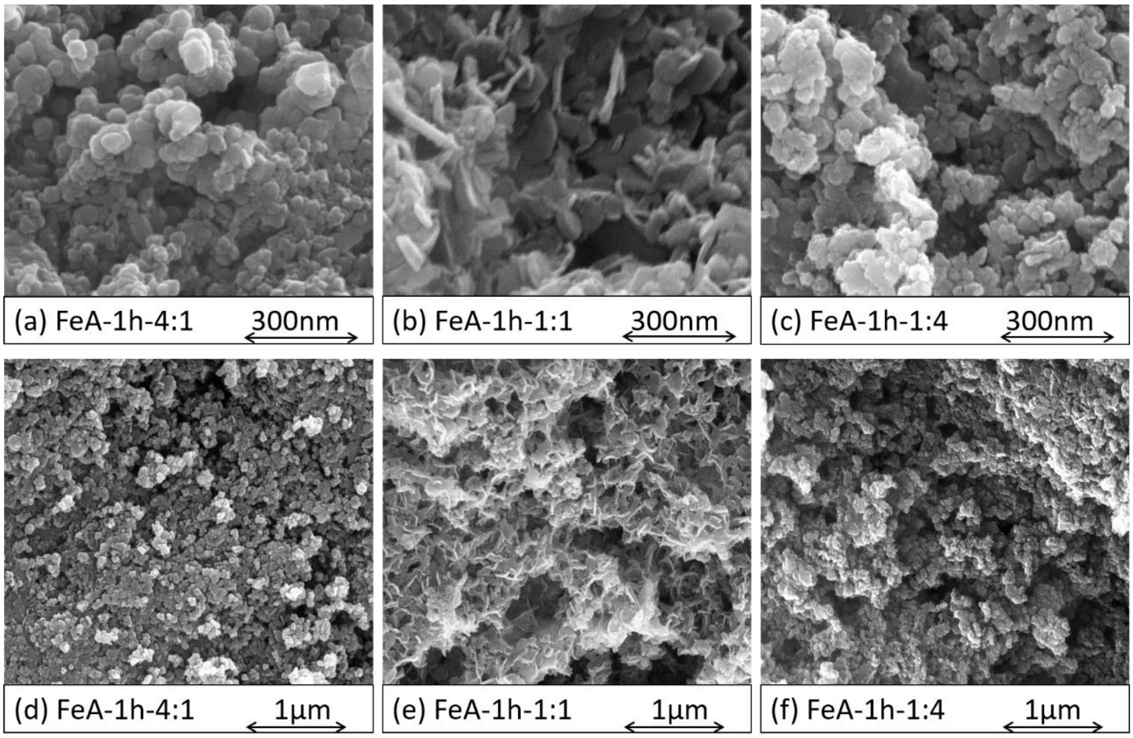

Figure S2. SEM images of the FeA synthesized for 1 hour with ratios R:M 4:1 (a and d), 1:1 (b and e) and 1:4 (c and f).

Reaction mechanism

The preparation of the reducing solution (composed of sodium carbonate and glyoxylic acid) in a basic media gives rise to the deprotonation of the glyoxylic acid, resulting in a transparent solution (Figure S3a) with a pH of 10.4 composed of oxalic and glycolic acid. On the other hand, the metallic precursor was prepared by dissolving iron chloride in water. In aqueous media, the iron ions are solvated resulting in a slightly orange solution (Figure S3b) with a pH value of 3.8. The mixture of these two solutions produces insoluble green complexes (Figure S3c).

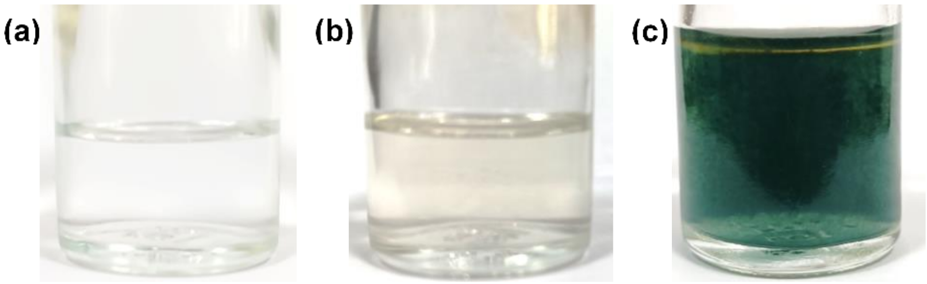

Figure S3. Photographs of the (a) reducing solution, (b) metallic solution, and (c) precursor mixture before starting the reaction.

Surface chemical composition

The surface elemental composition of the aerogels prepared for 4 hours was analyzed by XPS. The survey spectra obtained are presented in Figure S4, from which it can be confirmed that only iron, carbon, and oxygen are present in the aerogels. The data obtained from the deconvolution of the spectra of iron in the Fe 2p region are presented in Table S1.

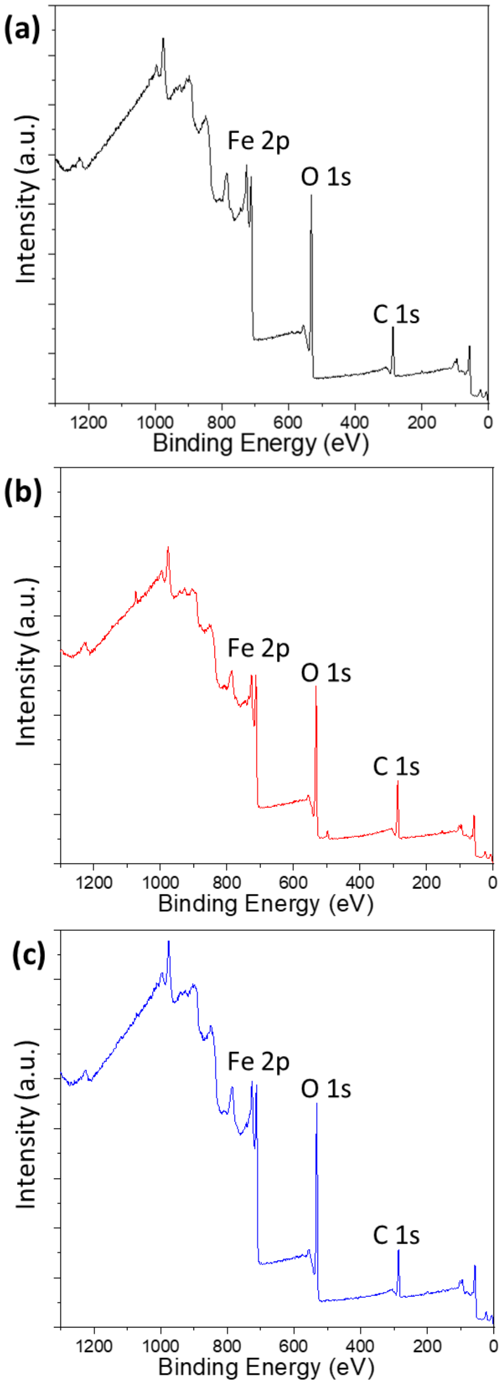

Figure S4. XPS survey corresponding to (a) FeA-4h-4:1, (a) FeA-4h-1:1 and (a) FeA-4h-1:4.

Table S1. Results obtained from the deconvolution of the high-resolution XPS spectra in the Fe 2p3/2 region*.

	Fe^{2+}			Fe^{3+}			Fe^{3+}			Surface			Satellite Fe^{3+}		
**Sample**	eV	FWHM	%	eV	FWHM	%	eV	FWHM	%	eV	FWHM	%	eV	FWHM	%
**FeA-1h-1:4**	710.7	2.5	28.3	712.4	2.5	13.9	714.0	2.5	7.0	715.6	2.5	2.3	719.2	5.8	16.2
**FeA-4h-4:1**	710.8	2.3	12.9	712.3	2.3	9.3	713.8	2.3	10.1	715.5	2.3	4.0	719.3	7.9	23.6
**FeA-4h-1:1**	710.9	2.1	25.3	712.4	2.1	14.7	713.9	2.1	7.5	715.5	2.1	3.1	719.4	5.8	16.6
**FeA-4h-1:4**	710.8	2.6	26.6	712.5	2.6	14.2	714.2	2.6	7.2	715.9	2.6	2.0	719.3	6.1	17.9

*The peaks of the 2p1/2 region are at 13.5 eV and for the p-subshell, the ratio of the 1/2:3/2 area is 1:2, so only the information of the 2p3/2 region is shown.

X-ray diffraction

Figure S5 shows the PDF used to identify the XRD phases of the iron aerogels.

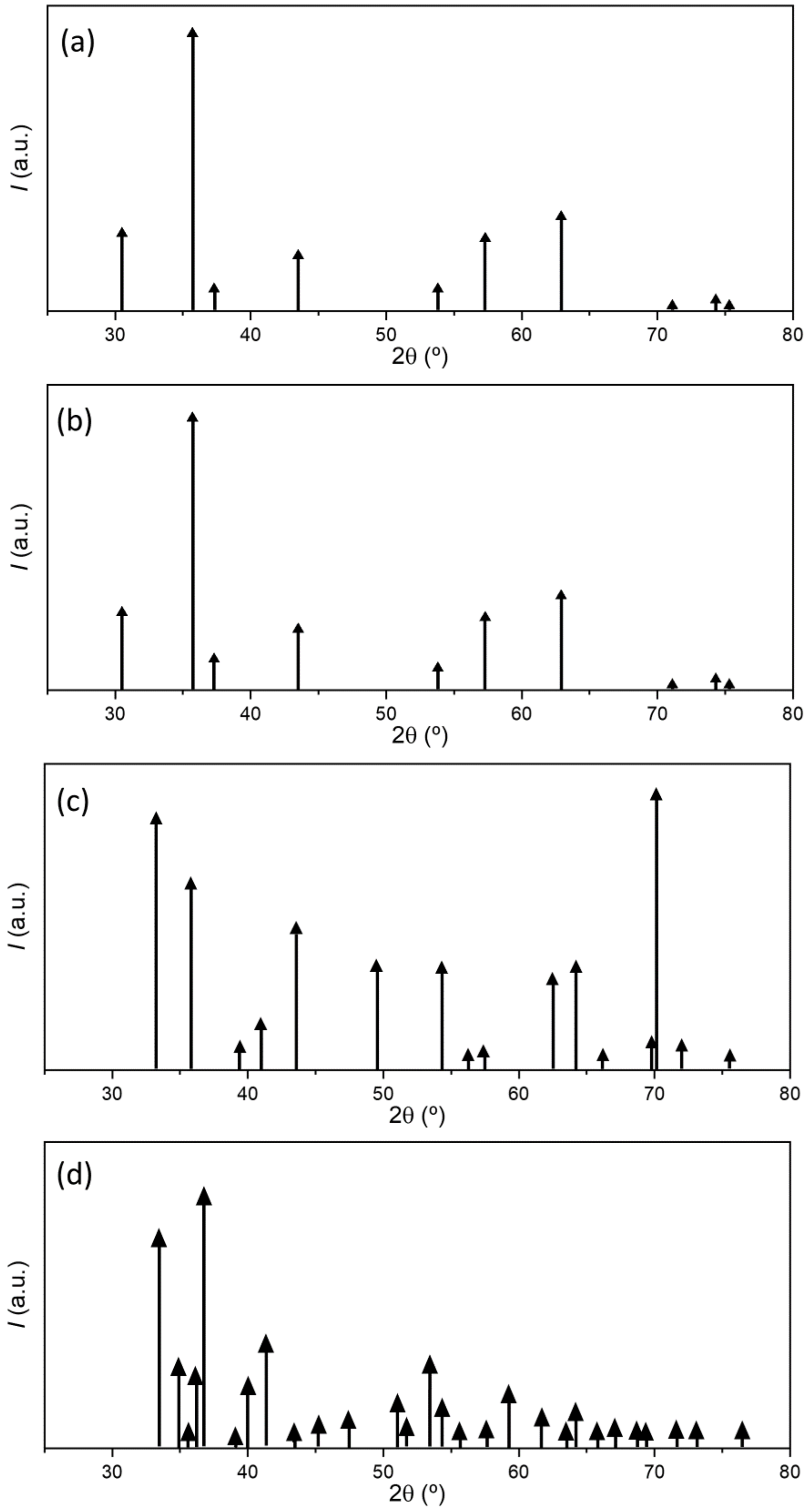

Figure S5. Standard XRD patterns for (a) magnetite (PDF no. 01-071-6337); (b) maghemite (JCPDS card number 04-0755); (c) hematite (ICDD card number 33-0664); and (d) goethite (ICDD card number 29-0713

The crystalline sizes of the iron aerogels are calculated using the Scherrer equation (Eq. S1), applied to the peak attributed to the (311) plane, which is the most intense peak of the diffractogram and closest to low angles, to avoid the splitting due to the contribution of the lines $K_{\alpha 1}$ and $K_{\alpha 2}$. The obtained data are shown in Table S2.

$$Lc=\lambda \cdot K/(\beta \cdot \cos(\theta)) \quad \text{(Eq S1)}$$

where L is the apparent particle size, λ is the characteristic wavelength of the X-ray tube used (λ(Cu)=1.5418 Å), K is the Scherrer constant dependent on the grain shape and the method used to estimate its size, β is the half-height width of the diffraction peak (in radians), and θ is the Bragg angle (in radians).

Table S2. Characteristic parameters obtained from XRD.

**Sample**	**Angle (º)**	**FWHM (º)**	**Intensity (a. u.)**	***d* (nm)**
Fe-1h-4:1	35.6	0.443	480	19
Fe-1h-1:1	35.4	0.203	850	41
Fe-1h-1:4	-	-	-	-
Fe-4h-4:1	35.8	0.516	300	16
Fe-4h-1:1	35.5	0.298	370	28
Fe-4h-1:4	-	-	-	-
Fe-8h-4:1	35.7	0.425	260	19
Fe-8h-1:1	35.5	0.331	750	25
Fe-8h-1:4	-	-	-	-

Magnetic response

The magnetic response evaluated by approaching a magnet and the difference in colour of the final iron aerogels are shown in Figure S6.

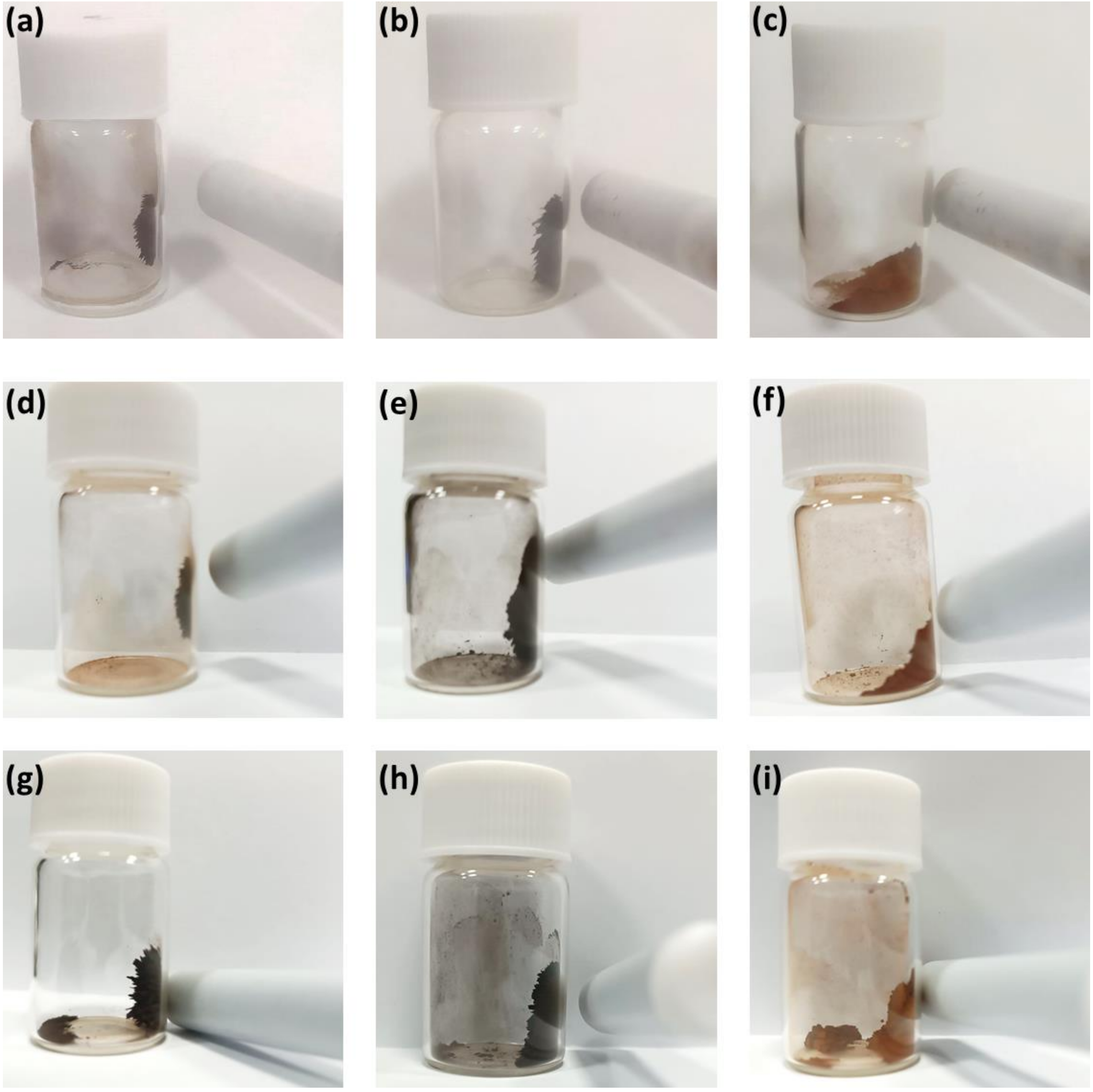

Figure S6. Photographs of the samples' response when approaching a magnet and the colour of FeA synthesized for 1 hour with ratios R:M (a) 4:1, (b) 1:1 and (c) 1:4, 4 hours with ratios R:M (d) 4:1, (e) 1:1 and (f) 1:4 and 8 hours with ratios R:M (g) 4:1, (h) 1:1 and (i) 1:4.
